# Individual differences in skill acquisition and transfer assessed by dual task training performance and brain activity

**DOI:** 10.1186/s40708-022-00157-5

**Published:** 2022-04-02

**Authors:** Pratusha Reddy, Patricia A. Shewokis, Kurtulus Izzetoglu

**Affiliations:** 1 School of Biomedical Engineering, Science and Health Systems, Drexel University, 3508 Market St Suite 100, Philadelphia, PA 19104 USA; 2 Nutrition Sciences Department—College of Nursing and Health Professions, Drexel University, 1601 Cherry St Free Parkway, Philadelphia, PA 19102 USA; 3 School of Education, 3401 Market Street 3rd Floor Suite 3000, Philadelphia, PA 19104 USA

**Keywords:** Human performance, Training, Individual differences, Functional brain imaging, Near-infrared spectroscopy, fNIRS, Skill acquisition, Transfer

## Abstract

Assessment of expertise development during training program primarily consists of evaluating interactions between task characteristics, performance, and mental load. Such a traditional assessment framework may lack consideration of individual characteristics when evaluating training on complex tasks, such as driving and piloting, where operators are typically required to execute multiple tasks simultaneously. Studies have already identified individual characteristics arising from intrinsic, context, strategy, personality, and preference as common predictors of performance and mental load. Therefore, this study aims to investigate the effect of individual difference in skill acquisition and transfer using an ecologically valid dual task, behavioral, and brain activity measures. Specifically, we implemented a search and surveillance task (scanning and identifying targets) using a high-fidelity training simulator for the unmanned aircraft sensor operator, acquired behavioral measures (scan, not scan, over scan, and adaptive target find scores) using simulator-based analysis module, and measured brain activity changes (oxyhemoglobin and deoxyhemoglobin) from the prefrontal cortex (PFC) using a portable functional near-infrared spectroscopy (fNIRS) sensor array. The experimental protocol recruited 13 novice participants and had them undergo three easy and two hard sessions to investigate skill acquisition and transfer, respectively. Our results from skill acquisition sessions indicated that performance on both tasks did not change when individual differences were not accounted for. However inclusion of individual differences indicated that some individuals improved only their scan performance (Attention-focused group), while others improved only their target find performance (Accuracy-focused group). Brain activity changes during skill acquisition sessions showed that mental load decreased in the right anterior medial PFC (RAMPFC) in both groups regardless of individual differences. However, mental load increased in the left anterior medial PFC (LAMPFC) of Attention-focused group and decreased in the Accuracy-focused group only when individual differences were included. Transfer results showed no changes in performance regardless of grouping based on individual differences; however, mental load increased in RAMPFC of Attention-focused group and left dorsolateral PFC (LDLPFC) of Accuracy-focused group. Efficiency and involvement results suggest that the Attention-focused group prioritized the scan task, while the Accuracy-focused group prioritized the target find task. In conclusion, training on multitasks results in individual differences. These differences may potentially be due to individual preference. Future studies should incorporate individual differences while assessing skill acquisition and transfer during multitask training.

**Supplementary Information:**

The online version contains supplementary material available at 10.1186/s40708-022-00157-5.

## Introduction

With recent advances in autonomy capability, we expect human–autonomy systems to be safe and efficient. However, the necessary and expected requirements in safety and efficiency have not yet been met. In fact, reports over the last decade in the fields of transportation, aviation, health care, and ergonomics have indicated human error as the largest contributing factor behind many severe accidents [1]. Increased information processing load and decision-making demands placed on the human operator because of the highly complex systems and task objectives have often been cited as primary reasons behind these accidents.

A common solution to ensure that an operator can handle such task loads or demands is to have them undergo an effective theory-based training program (i.e., cognitive load theory), which encourages schema construction and automation, so that performance is high. Importantly, the use of cognitive processes, such as working memory and attentional resources, while executing day-to-day tasks are not only reduced but freed up to allow for attendance to new tasks or any unforeseen events [2, 3]. In other words, investigating the effects of a training program consists of analyzing performance and correlates of mental load as a function of task characteristics. Moreover, such an analysis paradigm should consider individual differences while assessing training particularly on safety critical tasks due to operators being required to execute or attend to multiple tasks simultaneously [4].

Insight into training on multitasking can be derived from the dual task literature. This literature indicates that analysis of training on multitasks should consist of evaluating performance and correlates of mental load not only as a function of the task characteristics but also as a function of individual characteristics [5–8]. Firstly, task characteristics should include not just each task’s load but also the load generated from cross talk between or among tasks being learned. Inclusion of the interaction effect between tasks is especially important since tasks that share cognitive resources (e.g., digit and letter categorization tasks vs two digit categorization tasks) increase mental load and decrease performance than those that do not [9, 10]. Secondly, individual characteristics should include intrinsic (i.e., age, handedness, etc.), contextual (i.e., prior knowledge regarding the task, stress, priority, etc.), strategic (i.e., serial, parallel, semi-parallel), and personality (i.e., resistance to change, motivational tendencies, etc.) factors [11–14]. Lastly, individual characteristics arising from preference should also be included, especially when the multitask is being performed under high task load condition (e.g., weather changes) or when both tasks are classified as high priority [15–20]. Use of individual characteristics to improve training, so that skills can be acquired faster, transferred to new situations, and retained for an extended period, has been a major topic of interest in adaptive and personalized training [21]. This study aligns with this emerging incentive and aims to investigate whether accounting for these individual characteristics improves our understanding of multitask training effects, especially in ecologically valid task and novice operators.

While performance is commonly evaluated using behavioral measures, such as reaction time and accuracy, many techniques exist to measure workload, mental capacity, and mental effort. These techniques include subjective rating methods (i.e., NASA TLX, etc.), performance data from secondary tasks, and physiological sensors (i.e., eye tracking, galvanic skin response, etc.). Although such techniques have been used numerous times to assess workload and offer many advantages, they do not enable direct measurement of cognitive resources from the brain or real-time monitoring of the mental state of an individual while they are executing a task [22]. Additionally, they may not be able to identify changes in mental load as a function of individual characteristics. Therefore, there is a need to assess skill acquisition and transfer during training on an ecologically valid dual task using methods that measure cognitive resources directly.

Studies utilizing brain monitoring techniques, such as functional magnetic resonance imaging (fMRI) enable direct measurement of cognitive resources. These studies have indicated individual differences in brain activity as a result of individual characteristic (i.e., strategy, preference, etc.) [7]. Although fMRI studies have improved our understanding of the connection between brain and behaviors, their use of tasks that are not ecologically valid limits translation and repeatability of the results into field settings, especially those related to multitask training.

As an emerging interdisciplinary field, neuroergonomics has introduced methods needed to objectively assess skill acquisition and transfer in natural, everyday settings. This field is focused on understanding, evaluating, predicting, and improving key factors of human performance (such as workload, training, stress, and fatigue) in everyday settings via wearable brain-based technologies, such as functional near-infrared spectroscopy (fNIRS) [23]. fNIRS is a non-invasive and portable neuroimaging modality capable of continuously measuring correlates of brain activity from the cortex in ecologically valid environments. fNIRS functions under three principles: (1) increased neural activity leads to an increase in metabolic demands, which results in an increase in oxyhemoglobin (HbO) and deoxyhemoglobin (HbR) concentrations; (2) these hemoglobin chromophores have unique optical properties within the 700 to 900 nm wavelength; and (3) by examining the manner in which light passes through cortical tissue, concentrations of HbO and HbR can be calculated [24–26].

Over the last decade, fNIRS has been used extensively to assess workload, quantify mental capacity, and track training in both laboratory and field settings [22, 26–33]. Majority of these studies have focused on quantifying changes in brain activity within the prefrontal cortex (PFC), which is responsible for executive functions, such as working memory, attention, problem solving, decision making, response inhibition, planning, conflict resolution, and mental flexibility [34]. In summary, these studies have indicated three important takeaways: (1) additional task load leads to an increase in brain activity during both standard and complex tasks; (2) these brain activity changes acquired by fNIRS are complementary to behavioral metrics; (3) task practice decreases the extent or intensity of brain activity changes, particularly in the attentional and control areas while maintaining high behavioral or outcome performance. To a limited extent, fNIRS has also been used as a tool to conduct adaptive training [27, 35]. However, to our knowledge, fNIRS has not been used to assess individual difference in performance during dual task training.

In this paper, we investigated the effect of individual differences in skill acquisition and transfer during training on an ecologically valid dual task using behavioral and brain activity measures acquired from fNIRS. We selected an Unmanned Aerial System (UAS) operators’ search and surveillance task, as it is a dual task requiring an active role of attention and spatial working memory to ensure complete scanning of an assigned area and high accuracy of identifying and tracking targets. The duality of the task enabled the possibility of modeling of individual differences. We hypothesize that performance will vary across individuals and these changes will be reflected in fNIRS measures. Based on a previous investigation of skill acquisition using a similar task, we hypothesize that those participants who demonstrate an increase in their performance with practice across easy sessions will display a decrease in brain activity within task-specific PFC regions [36, 37]. Additionally based on a previous investigation of transfer, we hypothesize that those who reveal improvement in performance during easy sessions will presumably show transfer of skills during the hard sessions.

## Materials and methods

### Participants

Thirteen participants between the ages of 19 and 40 (22.92 ± 5.88 years) voluntarily consented to participate in an Institutional Review Board (IRB) approved study. Out of the 13 participants, nine were male and four were female. Recruited participants had no learning disability or sleep disorders, had either normal or corrected to normal vision, and had no prior experience with the simulator used in this study. However, participants had varying levels of overall experience playing three-dimensional (4.27 ± 6.07 h) and first-player three-dimensional (3.23 ± 4.42 h) games. Lastly, all participants were assessed as right-handed via use of the Edinburgh Handedness assessment (laterality index: 75.33 ± 20.05; and decile: 5.00 ± 3.58) [38].

### UAS Training Simulator

**Fig. 1:**
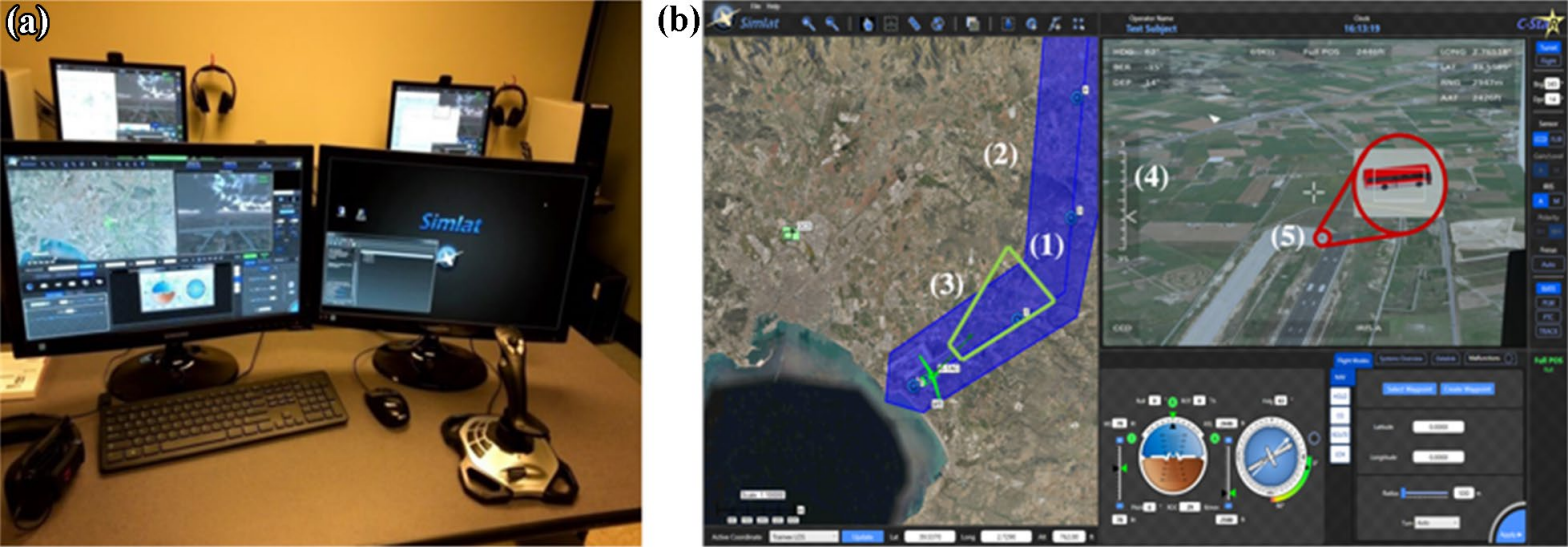
Unmanned aerial system training simulator. **A** The simulator allows for two trainees and one instructor. **B** The trainee screen is divided into map screen on the left and payload or sensor screen on the right. The map screen displays the route that the aircraft will fly along (1), the area where the scan and target find task are assigned (2), and what region the sensor screen is capturing (3). The payload screen displays real-time visual of the landscape being looked at with feedback regarding the zoom level (4). This screenshot also shows how the target (5), a red civilian bus, looks like from a distance and when it is being tracked at a zoom angle of 3°

A UAS simulator training setup (C-STAR, Simlat Inc., Miamisburg, Ohio) was used in this study, as it allowed for close implementation of a real operator work environment and presented a realistic representation of the daily task. This simulator is currently being used to support over 80 UAS training centers across 30 countries and has been used in previous studies [37]. The simulator permits for a two-trainee and one-instructor setup, training on sensor operation and pilot tasks, and for the instructor to manually or automatically preset ‘emergency’ situations that the operator(s) might encounter (e.g., such as developing inclement weather conditions, and equipment failure). In the present study, the simulator was used in a single-instructor and trainee configuration (see Fig. 1A), where the flight was auto-piloted and sensor operator’s search and surveillance task was implemented. The trainee’s simulator’s screen is partitioned into a map and payload portion. The map portion displays the flight path and assigned region on the landscape where the tasks need to be executed (blue line and shaded blue region in Fig. 1B), provides real-time feedback on the location of the aircraft relative to the flight path, and indicates what portion of the overall map or shaded blue region is under the camera’s field of view (FOV; green polygon in Fig. 1B). The sensor operator’s screen displays the actual landscape under the camera's FOV, and the zoom level associated with the FOV. Additional scopes regarding the engine and the flight are provided below the payload screen; however, these functionalities were disabled in this study.

### fNIRS Instrumentation

**Fig. 2:**
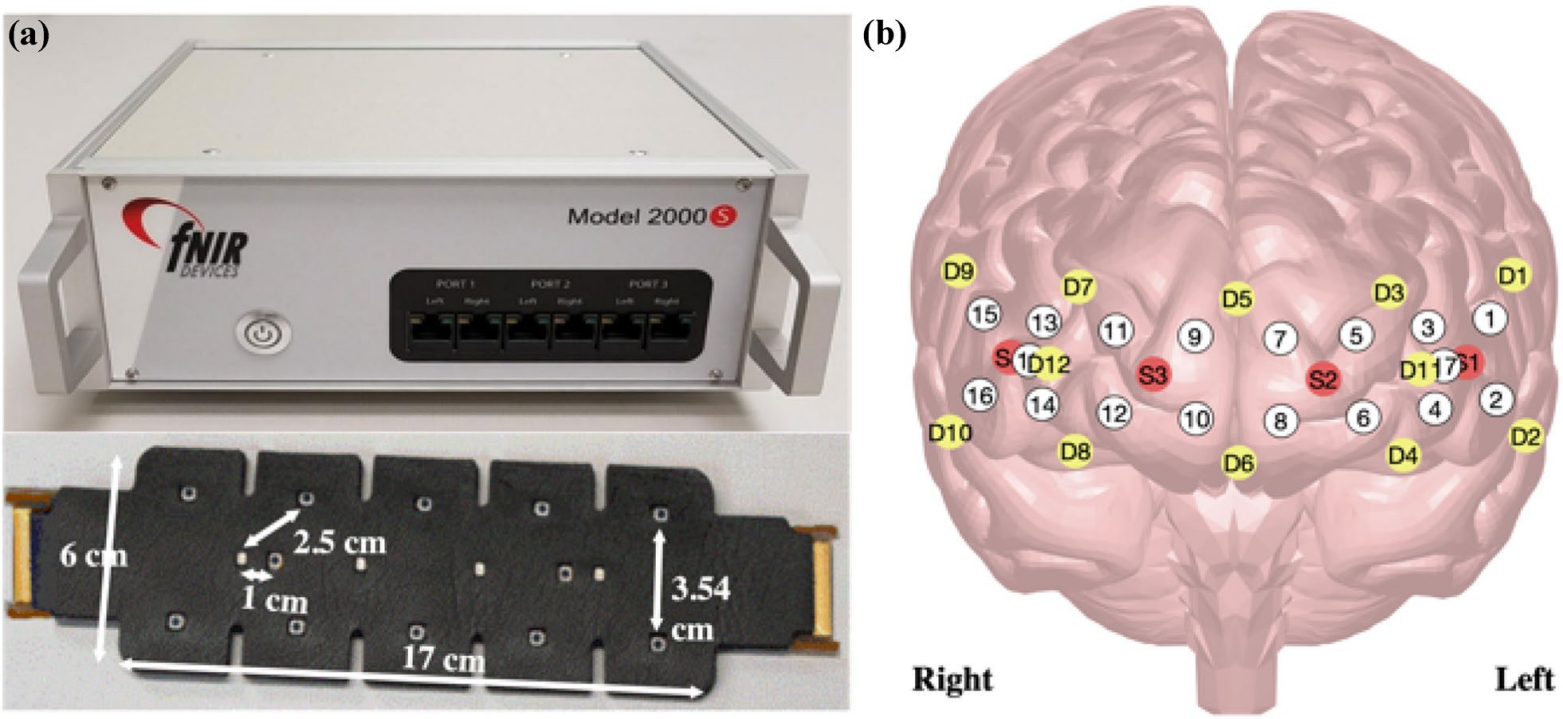
Functional near-infrared spectroscopy system used to collect relative changes in oxyhemoglobin and deoxyhemoglobin concentrations. **A** The data acquisition and sensor used. The sensor allowed for measurement of cerebral and extracerebral activities using long (2.5 cm) and short (1 cm) source detector separation (SDS) channels. **B** The location of sources or light emitting diodes (red circles), detectors or photodiodes (yellow circles), and channels (white circles) on the prefrontal cortex. Channels 1 through 16 represent cerebral measurements, while channels 17 and 18 represent extracerebral measurements

Hemodynamic changes from PFC were monitored using the fNIR Imager 1200 (fNIR Devices LLC, Potomac, MD) (see Fig. 2A). The system operates at a sampling frequency of 10 Hz and measures light intensity during ambient (when no light is shone), 750, and 830 nm wavelengths. The sensor has four surface mount light emitting diodes (red circles in Fig. 2B), and twelve silicone photodiodes with integrated transimpedance preamp (yellow circles in Fig. 2B). Ten of the twelve detectors are located 2.5 cm away from each source and enable measurement of cerebral activity from 16 different locations (white circles labeled 1 through 16 in Fig. 2B). The remaining two detectors are located 1 cm away from the middle two sources and allow measurement of extracerebral activity from two different locations (white circles labeled 17 and 18 in Fig. 2B).

### Experimental protocol

**Fig. 3:**
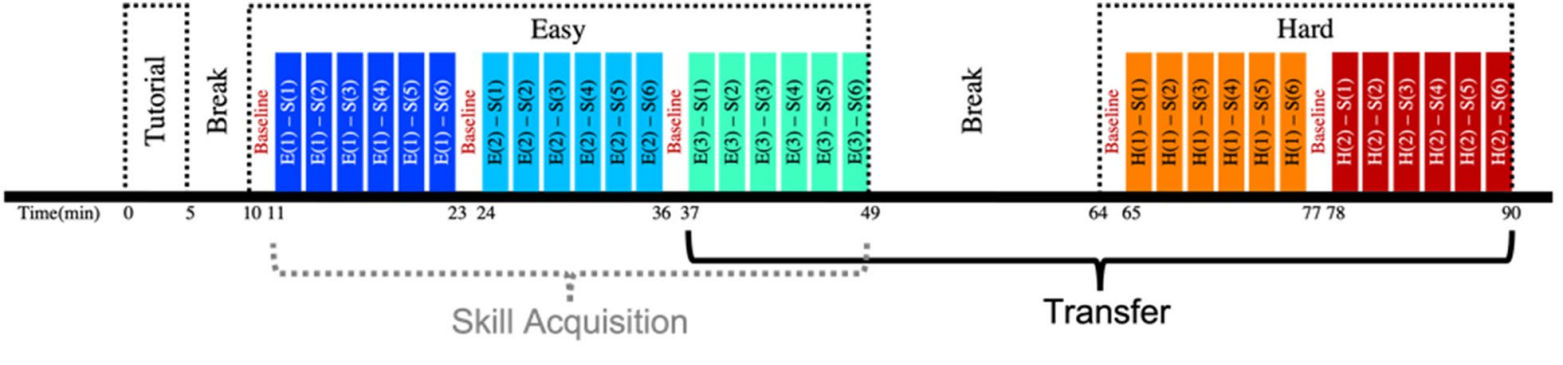
Experimental protocol began with a tutorial session followed by easy and hard sessions, respectively. Easy sessions consisted of three similar scenarios that occurred at 11:00 AM (simulator time) and were randomly administered. Hard sessions consisted of two different scenarios that occurred at 8:00 PM or 6:00 AM and were also randomly administered. Each scenario was approximately 12 min long and consisted of six subareas that each lasted 2 min. Within each of these subareas’ participants were required to scan the assigned area and find the target

Each participant underwent a tutorial session, followed by three easy sessions and two hard sessions (see Fig. 3). The tutorial session lasted five minutes, during which the participants were shown how to utilize the joystick and the computer mouse to navigate across map and payload screens, how to lock and track a target, and what constituted as proper scanning and target find behaviors, which were defined as completely scanning the assigned region and tracking the target (red civilian bus as shown on the screen in Fig. 1B) at or below a zoom level of 15° for at least 3 s. After instructions were given, participants were allowed to practice utilizing the screens and equipment to execute the tasks for the remainder of the tutorial session. After the tutorial session was over, participants were told to prioritize both tasks equally. The easy and hard sessions were approximately 12.5 min in duration, and all had unique flight paths with inimitable target placements. A 15-min break was given between the easy and hard sessions, during which time the fNIRS was taken off. The primary difference between easy and hard sessions was that the easy sessions occurred at a simulation time of 11:00AM, while the hard sessions occurred at 8:00PM or 6:00AM. To ensure that the bias was minimized, easy, and hard scenarios were randomized during their specific time periods. Each session consisted of six subareas, which each lasting 2 min on average. A 10-s gap was programmed between each subarea, allowing participants to re-adjust their camera settings. Based on this experimental protocol, the independent variable was session, while the subareas were repetitive measures. The changes in easy sessions were used to assess skill acquisition, while changes from last easy to first hard session and between hard sessions were used to assess transfer.

### Preprocessing of fNIRS data

fNIRS signals are often confounded by factors, such as motion artifacts, head movement, systemic physiological changes, instrumentation, and environment noises. To extract neural activity-related signals, the following methods were applied. Channels that were saturated (> 4500), had high dark current values (> 200), or had high correlations between wavelength and ambient measurements (*r* > 0.7) were removed from further analysis [39, 40]. Abrupt spikes were removed via wavelet-based motion artifact removal [41]. Low-frequency drifts and high-frequency noise associated with respiration and cardiac functions were removed via a high- and low-pass finite impulse response filters with cut-off frequencies at 0.005 and 0.1 Hz [42]. Optical density data were then converted into the relative concentration changes of HbO and HbR using modified Beer Lambert law [25].

### Dependent variables

The Performance Analysis & Evaluation module of the simulator quantifies trainee’s performance in terms of scan (area covered within the assigned ROI area), not scan (area not covered with the assigned ROI area), and over scan (area covered outside of the assigned ROI area) percentage. An example of these measures extracted from a subarea of an individual is shown in Additional file 1: Figure S1. The module also records cameras zoom level and a logical index (0 or 1) representing when target is in FOV or is not in FOV every microsecond. Using this information, we set accuracy to be ‘1’ if the target was in FOV, and the scan occurred at a zoom level at or below 15°. This criterion was provided by the field expert. In subareas that did not have targets, accuracy was set to ‘1.’ Since accuracy may be affected by performance from the previous subarea, an adaptive target find (AdpTF) score was calculated by dividing accuracy by the number of the subarea.

In alignment with the approach previously reported by Izzetoglu et al., average HbO and HbR measures between 15 s after the start of the subarea and 15 s before the end of the subarea were extracted from each channel [43]. This was performed as the tasks here followed each other continuously to maintain ecological validity, i.e., no resting periods in between, and to wash out any effect from the preceding task's hemodynamic response that could be carried over to the present task.

To simultaneously evaluate behavioral and hemodynamic measures, relative efficiency (RE) and relative involvement (RI) measures were calculated using Eqs. 1 and 2 [32, 44]. In the equations, P represents standardized performance score (e.g., scan, or AdpTF), while M represents standardized mental effort score (HbO and HbR). HbR measures were multiplied by −1 to fit within the efficiency definition, because, unlike HbO, HbR measures are expected to be negative and increase with practice.

1 $${\text{Relative}}\,{\text{Efficiency}} = \left( {P - M} \right)/\sqrt 2 ,$$

2 $${\text{Relative}}\,{\text{Involvement}} = \left( {P + M} \right)/\sqrt 2 .$$

### Grouping based on individual differences

Subject matter field experts use scan behavioral performance measure as an indicator of expertise development. Improvement in scanning suggests good performance, while deterioration indicates poor performance. Therefore, median measures of scan performance across subareas were extracted per subject and changes in the median scan performance measures across easy sessions were estimated individually for each subject using linear regression in R. Individuals that displayed a positive slope were placed under one group, while those with negative slope were placed in another group.

### Statistics

Due to a hierarchical nesting structure and the presence of missing data (determined to be missing at random), linear mixed effects regression (LMER) modeling was used. Firstly, models generated investigated the incorporation of individual differences factor (Group) in improving modeling fitness of behavioral (4 measures: scan, not scan, over scan, AdpTF) and mean fNIRS (32 measures: HbO and HbR from channels 1 through 16) measures against models without individual differences factor (Group). Secondly, to investigate changes in performance and mental effort during skill acquisition and transfer separately, Eqs. 3 and 4 were used to evaluate the main and interaction effects of Group (Group 1 and Group 2), Session (Easy 1—E1, Easy 2—E2, Easy 3—E3, and Hard 1—H1 and Hard 2—H2) and AdpTF on behavioral (3 measures: scan, not scan, and over scan), and mean fNIRS (32 measures: HbO and HbR from channels 1 through 16) measures, respectively. The interaction between Group, Session, and AdpTF was incorporated as a fixed effect to the models being investigated, to evaluate the cross talk between scan and target find tasks. The model investigating behavioral measures used a random intercept design, while the model investigating mean fNIRS measures used non-correlated random intercept and slope design. The random slope term (0 + Short | ID) when evaluating mean fNIRS measures was used to separate task-induced extracerebral activity from that related to cerebral activity, which was shown to be significant in another study using the same dataset [45]. Lastly, to investigate performance and mental effort simultaneously, Eq. 5 was used to evaluate the main and interaction effects of Group and Session on RE and RI measures calculated from following behavioral (Scan and AdpTF) and mean fNIRS measures (channels largely effected by the dual task). Like Eq. 4, a random slope design was used to account for extracerebral RE and RI.

3 $${\text{DV}} \sim 1 + {\text{Group}} + {\text{Group}}:{\text{Session}} + {\text{Group}}:{\text{Session}}:{\text{AdpTF}} + \left( {1|{\text{ID}}} \right),$$

4 $${\text{DV}} \sim 1 + {\text{Group}} + {\text{Group}}:{\text{Session}} + {\text{Group}}:{\text{Session}}:{\text{AdpTF}} + \left( {1|{\text{ID}}} \right) + \left( {0 + {\text{Short}}|{\text{ID}}} \right),$$

5 $${\text{DV}} \sim 1 + {\text{Group}} + {\text{Group}}:{\text{Session}} + \left( {1|{\text{ID}}} \right) + \left( {0 + {\text{Short}}|{\text{ID}}} \right).$$

Significance of fixed effect terms was evaluated using likelihood ratio tests, where the full effects model was compared against a model without the effect in question. Maximum likelihood estimation was used to conduct likelihood ratio tests, while restricted maximum likelihood was used to evaluate post hoc comparisons. If an interaction term was significant, then planned comparisons were performed between the same sessions of different groups (e.g., Attention-focused vs Accuracy-focused of E1) to investigate between-subject differences and between sessions of the same group (e.g., Attention-focused: E1 vs E3) to investigate within-subject differences. Specifically, the within-subject differences enabled investigation of skill acquisition (E1 vs E3) and transfer (E3 vs H1 or H1 vs H2). A total of 10 comparisons were conducted per each dependent variable. Homogeneity of variance, and normality of residuals and random effects were conducted using visual inspection. If model predictions showed heteroscedasticity or non-normal distribution, then log10 transformations were performed on the response variables. Satterthwaite approximation of degrees of freedom was used in post hoc analyses [46]. For all statistical analyses, the level of significance was set at  α = 0.05. Adjustments using false discovery rate (FDR) were made on *p* values to account for Type I error inflation per dependent variable. Cohen’s *d* was used to examine post hoc effects [47]. *d* of 0.2 is considered a small effect, while 0.5 and ≥ 0.8 represent medium and large effects, respectively. All statistical analyses were conducted in R (R Core Team, 2019) using *lme4, lmerTest,* and *emmeans* functions [48–50].

## Results

### Effect of incorporating individual differences as a fixed factor while evaluating performance and mean fNIRS measures

**Fig. 4:**
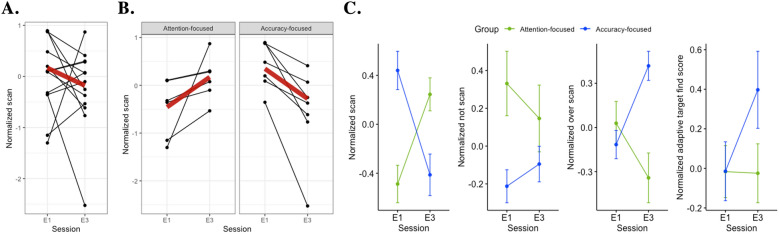
Overall and subject-specific changes in behavioral measures across training sessions. **A** Changes in scan measures from easy session 1 (E1) to easy session 3 (E3). **B** Six individuals displayed an increasing change in scan measure from E1 to E3, while seven displayed a decreasing behavior. **C** Mean changes in scan, not scan, over scan, and adaptive target find measures. Black dots represent mean scan measures across subareas per subject and session. Colored dots and associated error bars represent mean and standard error of mean across subareas and subject

Per subject changes in scan measures from E1 to E3 prior to grouping displayed large variability in slope change (see Fig. 4A). Applying grouping based on individual differences in scan performance resulted in six subjects falling into a group where scan measures increased from E1 to E3, and seven subjects falling into a group where scan measures decreased (see Fig. 4B). Effect of this grouping on other behavioral measures indicated that individuals who improved in their scan performance also improved in their not scan and over scan performance (see blue lines in first three columns of Fig. 4C). However, these individuals displayed no change in their target find performance (see blue line in last column of Fig. 4C). Alternatively, the group that showed depreciation in scan performance also showed depreciation in not scan and over scan performances, while improving in target find performance (see red lines in Fig. 4C). Therefore, for the rest of the paper those who improved in scan performance will be referred to as Attention-focused group, while those who improved in target find performance will be referred to as Attention-focused group. As shown in Table 1, significant improvement in goodness of fit was observed for all behavioral measures when individual differences factor “Group” was added to the model. Similarly significant improvement in goodness of fit was also observed for mean fNIRS measures in most channels, especially for HbR (see Table 2).

**Table 1:** Comparison of goodness of fit between models with and without Group as fixed factor for behavioral measures

Dependent variable	Individual differences	Comparison against null model			Comparison between models	
		Log likelihood	${\chi }^{2}$	*p* value	${\chi }^{2}$	*p* value
Scan	No	− 526.10	12.00	0.017	33.40	< 0.001
	Yes	− 509.41	45.39	< 0.001		
Not scan	No	− 502.56	12.33	0.015	14.86	0.011
	Yes	− 495.13	26.32	0.001		
Over scan	No	− 524.71	1.73	0.786	19.50	0.002
	Yes	− 514.96	20.09	0.010		
Adaptive target find	No	40.42	5.39	0.250	13.45	0.020
	Yes	47.15	12.41	0.134		

The following models were compared: 1 + (1 | ID) vs 1 + Session + (1|ID), 1 + Group + (1 | ID) vs 1 + Group + Group: Session + (1|ID), 1 + Session + (1 | ID) vs 1 + Group + Group: Session + (1|ID). For models without and with individual differences, the number parameters were 7 and 12, and degrees of freedom were 4 and 8, respectively. Degrees of freedom for comparison between models were 5

**Table 2:** Comparison of goodness of fit between models with and without group as fixed factor for mean fNIRS measures from a subset of channels

Dependent variable		Individual differences	Comparison against null model			Comparison between models	
Channel	Biomarker		Log likelihood	${\chi }^{2}$	*p* value	${\chi }^{2}$	*p* value
2	HbO	No	− 341.48	25.79	< 0.001	30.21	< 0.001
		Yes	− 351.28	72.57	< 0.001		
	HbR	No	− 326.38	55.01	< 0.001	7.98	0.157
		Yes	− 347.29	77.04	< 0.001		
7	HbO	No	− 380.94	37.02	< 0.001	42.39	< 0.001
		Yes	− 400.75	14.61	< 0.001		
	HbR	No	− 359.75	79.40	< 0.001	28.93	< 0.001
		Yes	− 386.29	43.49	< 0.001		
11	HbO	No	− 389.93	16.79	< 0.001	6.53	0.258
		Yes	− 452.77	39.11	< 0.001		
	HbR	No	− 386.66	23.31	< 0.001	30.55	< 0.001
		Yes	− 437.50	69.12	< 0.001		
12	HbO	No	− 406.72	16.39	< 0.001	16.32	0.006
		Yes	− 392.65	67.45	< 0.001		
	HbR	No	− 398.56	30.14	< 0.001	42.39	< 0.001
		Yes	− 371.46	109.79	< 0.001		
14	HbO	No	− 396.43	32.47	< 0.001	6.40	0.269
		Yes	− 399.21	53.79	< 0.001		
	HbR	No	− 393.23	38.50	< 0.001	32.32	< 0.001
		Yes	− 383.04	86.11	< 0.001		

The following models were compared: 1 + (1 | ID) + (0 + Short | ID) vs 1 + Session + (1 | ID) + (0 + Short | ID), 1 + Group + (1 | ID) + (0 + Short | ID) vs 1 + Group + Group: Session + (1 | ID) + (0 + Short | ID), 1 + Session + (1 | ID) + (0 + Short | ID) vs 1 + Group + Group: Session + (1 | ID) + (0 + Short | ID). For models without and with individual differences, the number parameters were 8 and 13, and degrees of freedom were 4 and 8, respectively. Degrees of freedom for comparison between models were 5

### Effect of Group, Session, and Adaptive target find score on behavioral measures

Interaction between Group and Session was significant for scan ($\chi^{2}$(8) = 45.39, *p* < 0.001), not scan ($\chi^{2}$(8) = 26.32, *p* = 0.001), and over scan ($\chi^{2}$(8) = 20.09, *p* = 0.010) measures (see Additional file 1: Figure S2). Post hoc testing for interaction between Group and Session revealed significant differences only for scan measures. Specifically, comparisons between Groups per Session revealed significant differences only in easy session 1 (adj.*p* = 0.022, *d* = − 1.07), where the Accuracy-focused group had superior scanning than the Attention-focused group. Pairwise comparisons between sessions within the Attention-focused group indicated significant increases in scanning from easy session 1 to easy session 3 (adj.*p* = 0.002, *d* = − 0.84). Comparisons within the Accuracy-focused group indicated significant decreases in scanning from easy session 1 to easy session 3 (adj.*p* = 0.001, *d* = 0.92).

Interaction between Group, Session, and AdpTF was significant only for over scan ($\chi^{2}$(10) = 23.16, *p* = 0.010) measures. However, post hoc comparisons revealed no significant differences between Groups per Session or between Sessions per Group.

### Effect of Group, Session, and Adaptive target find score on mean fNIRS measures

**Fig. 5:**
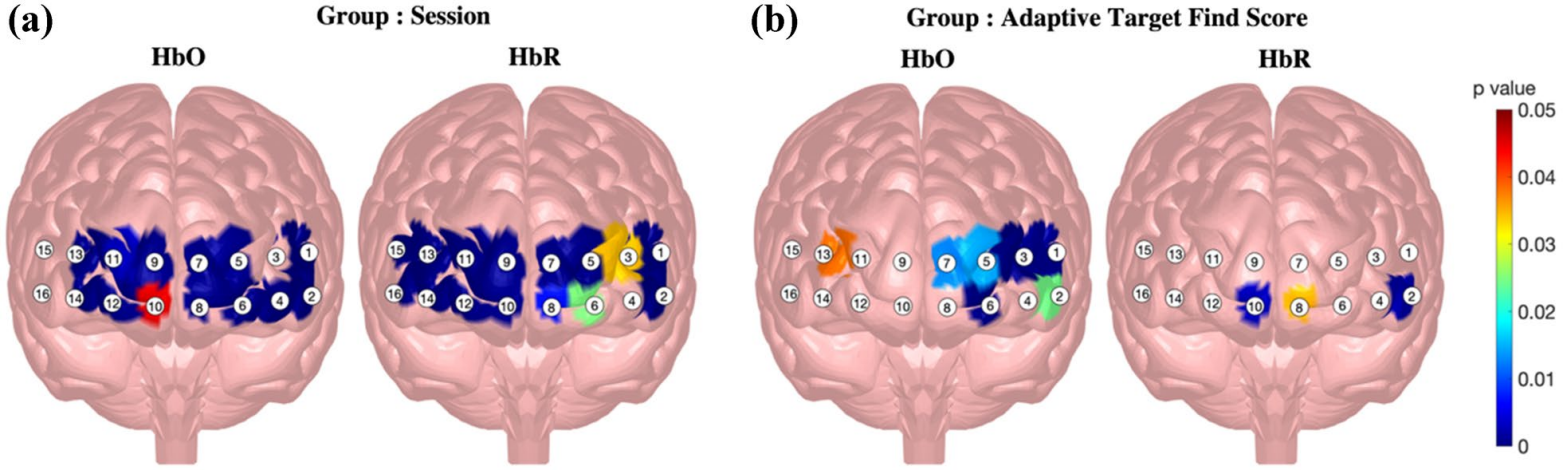
Significance of changes in mean fNIRS measures per channel and biomarker. **A** Interactions effects between Group and Session. **B** Interaction effects between Group, Session and Adaptive target find score. Channels with overlayed coloring indicate significance of the associated term at α less than 0.05

Interaction between Group and Session was significant across most channels, with only channels 3, 4, 15, and 16 displaying no significant effects across both fNIRS measures (see Fig. 5A). Largest effects were observed in channel 12 (HbO: $\chi^{2}$(8) = 30.14, *p* < 0.001; HbR: $\chi^{2}$(8) = 109.79, *p* < 0.001), followed by channels 2 (HbO: $\chi^{2}$(8) = 55.01, *p* < 0.001; HbR: $\chi^{2}$(8) = 77.04, *p* < 0.001), 14 (HbO: $\chi^{2}$(8) = 38.50, *p* < 0.001; HbR: $\chi^{2}$(8) = 86.11, *p* < 0.001), 7 (HbO: $\chi^{2}$(8) = 79.40, *p* < 0.001; HbR: $\chi^{2}$(8) = 43.49, *p* < 0.001), and 11 (HbO: $\chi^{2}$(8) = 23.31, *p* = 0.003; HbR: $\chi^{2}$(8) = 69.12, *p* < 0.001).

Evaluation of post hoc comparisons between Groups per Session for “Group: Session” term revealed significant activity in channels 2, 5, 7, 9, 12, 13, and 15. However, only results from channels 2, 7, and 12 are reported below, as they depicted the largest effects. In E1, significant differences were observed only in channel 2 (HbR: adj.*p* = 0.045, *d* = − 0.97), with larger mental effort exerted by Attention-focused group. In E3, significant differences were observed in channels 2 (HbR: adj.*p* = 0.025, *d* = − 1.15), 7 (HbO: adj.*p* = 0.004, *d* = 1.61), and 12 (HbR: adj.*p* = 0.026, *d* = 1.07), with Attention-focused performers having greater activity in channels 2 and 7, and Accuracy-focused performers having greater activity in channel 12. In H1, activity was significant only in channel 12 (HbR: adj.*p* = 0.045, *d* =  –0.94), with activity being dominant in the Attention-focused group. Lastly, in H2, activity was not significant in any channel.

Evaluation of pairwise comparisons between Sessions per Group for “Group: Session” term indicated significant activity across multiple comparisons within channels 2, 5, 7, 9, 11, 12, 13, and 14. However, only results from channels 2, 7, 11, 12, and 14 are reported below, as they signify the largest effects (for results from other channels refer to Additional file 1: Figure S3). In Attention-focused group, activity from (i) E1 to E3 increased in channel 7 (HbO: adj.*p* = 0.031, *d* = −0.74; HbR: adj.*p* = 0.008, *d* = 0.91), and decreased in channels 2 (HbR: adj.*p* < 0.001, *d* = −1.70), 11 (HbO: adj.*p* = 0.004, *d* = 1.05), 12 (HbR: adj.*p* < 0.001, *d* = −1.60), and 14 (HbR: adj.*p* < 0.001, *d* = −1.53); (ii) E3 to H1 decreased in channel 7 (HbO: adj.*p* = 0.049, *d* = 0.64), and increased in channels 11 (HbO: adj.*p* = 0.004, *d* = −0.95) and 12 (HbR: adj.*p* < 0.001, *d* = 1.02); (iii) H1 to H2 increased in channels 12 (HbO: adj.*p* = 0.002, *d* = −0.93; HbR: adj.*p* = 0.026, *d* = 0.60) and 14 (HbO: adj.*p* < 0.001, *d* = −1.18; HbR: adj.*p* = 0.007, *d* = 0.79). Alternatively, in Accuracy-focused group, activity from (i) E1 to E3 decreased in channels 2 (HbR: adj.*p* < 0.001, *d* =  − 1.88), 7 (HbO: adj.*p* < 0.001, *d* = 1.31; HbR: adj.*p* = 0.001, *d* = −1.13), 11 (HbR: adj.*p* < 0.001, *d* = −1.44), 12 (HbR: adj.*p* < 0.001, *d* = −0.97), 13 (HbO: adj.*p* = 0.010, *d* = 0.87; HbR: adj.*p* = 0.009, *d* = −0.79), and 14 (HbO: adj.*p* = 0.031, *d* = −0.74; HbR: adj.*p* = 0.008, *d* = 0.91); (ii) E3 to H1 increased in channels 2 (HbO: adj.*p* < 0.001, *d* = −1.67; HbR: adj.*p* = 0.042, *d* = 0.64) and 7 (HbO: adj.*p* < 0.001, *d* =  − 1.86), and decreased in channel 12 (HbR: adj.*p* < 0.001, *d* = −0.98); (iii) H1 to H2 decreased in channel 2 (HbO: adj.*p* = 0.001, *d* = 1.22; HbR: adj.*p* = 0.016, *d* = 0.80), and increased in channel 12 (HbR: adj.*p* = 0.002, *d* = 0.78).

Post hoc analysis associated with “Group: Session: AdpTF” indicated significant comparisons within channels 1, 2, 6, and 10. Only channel 1 displayed significant changes in relationship between AdpTF and brain activity across Groups per Session. Specifically, a significant difference was observed during E1 (HbO: adj.*p* = 0.037, *d* = 4.157), where Accuracy-focused performers displayed a negative relationship, while Attention-focused performers displayed a positive relationship. No comparisons across sessions per Attention-focused were significant. Alternatively, in Accuracy-focused group significant changes in relationship between AdpTF and brain activity were observed (i) from E1 to E3 in channels 1 (HbO: adj.*p* < 0.001, *d* = −5.22), 2 (HbR: adj.*p* = 0.001, *d* = 6.11), and 10 (HbR: adj.*p* = 0.025, *d* = −0.89), where relationship moved from negative to positive in channels 1 and 2, and from positive to negative in channel 10; (ii) from E3 to H1 in channels 2 (HbR: adj.*p* = 0.007, *d* = −1.93) and 6 (HbO: adj.*p* = 0.001, *d* = −2.19), where relationship moved from positive to negative and negative to positive, respectively; (iii) from H1 to H2 in channels 8 (HbR: adj.*p* = 0.018, *d* = 5.39) and 10 (HbR: adj.*p* = 0.010, *d* = 5.83), where relationship moved from positive to negative.

### Effect of Group and Session on relative efficiency and relative involvement measures

**Fig. 6:**
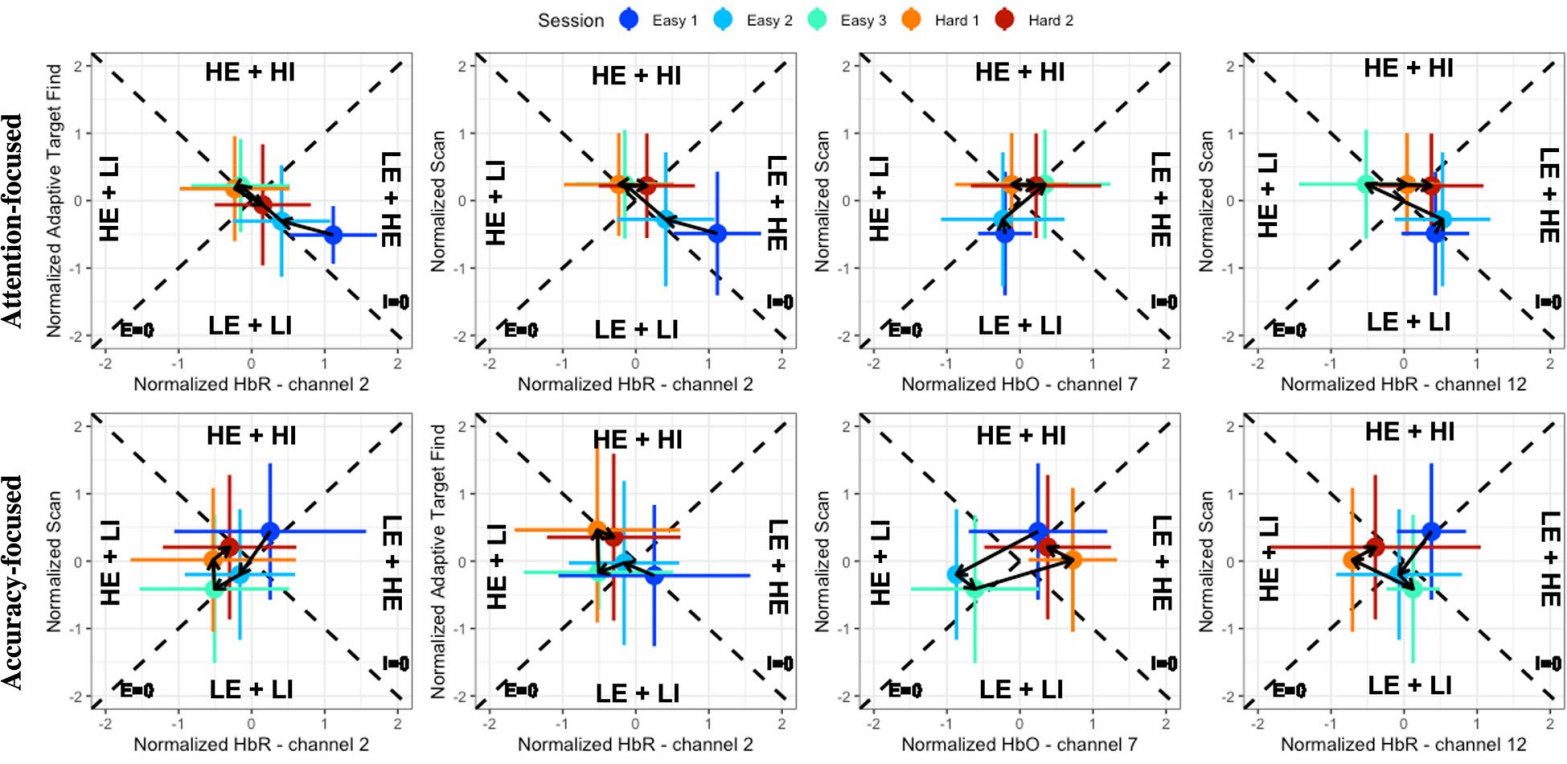
Changes in efficiency and involvement based on adaptive target find and scan performance and fNIRS measures from channels 2, 7, and 12 per Group and Session. The four quadrants generated by the efficiency (*E*) = 0 and involvement (*I*) = 0 lines represent combination of high efficiency (HE) or low efficiency (LE) and high involvement (HI) or low involvement (LI). Circles on the graphs reflect mean, while error bars represent standard deviation

Significant interaction effects of Group and Session were observed on RE and RI measures extracted from behavioral (scan) and mean fNIRS measures from channels 2 (RE—HbO: ${\chi }^{2}$(8) = 41.83, *p* < 0.001; HbR: ${\chi }^{2}$(8) = 51.78, *p* < 0.001; RI—HbO: ${\chi }^{2}$(8) = 52.04, *p* < 0.001; HbR: ${\chi }^{2}$(8) = 61.39, *p* < 0.001), 7 (RE—HbO: ${\chi }^{2}$(8) = 66.69, *p* < 0.001; HbR: ${\chi }^{2}$(8) = 26.10, *p* = 0.001; RI—HbO: ${\chi }^{2}$(8) = 79.57, *p* < 0.001; HbR: ${\chi }^{2}$(8) = 56.30, *p* < 0.001), and 12 (RE—HbO: ${\chi }^{2}$(8) = 34.84, *p* < 0.001; HbR: ${\chi }^{2}$(8) = 76.74, *p* < 0.001; RI- HbO: ${\chi }^{2}$(8) = 30.60, *p* < 0.001; HbR: ${\chi }^{2}$(8) = 30.68, *p* < 0.001) (see last three columns in Fig. 6).

Post hoc comparison of RE and RI between groups per session during E1 revealed significantly higher RE in Accuracy-focused group for channel 2 (HbR: adj.*p* = 0.004, *d* = −1.47) and higher RI in channel 7 (HbR: adj.*p* = 0.007, *d* = −1.37). During E3, Attention-focused group had higher RE in channel 12 (HbR: adj.*p* = 0.039, *d* = 1.09), and higher RI in channels 2 (HbR: adj.*p* = 0.009, *d* = 1.33) and 7 (HbO: adj.*p* = 0.005, *d* = 1.50; HbR: adj.*p* = 0.013, *d* = 1.22). Alternatively, Accuracy-focused group had higher RE in channels 2 (HbR: adj.*p* = 0.042, *d* = -–1.02) and 7(HbO: adj.*p* = 0.030, *d* = −1.37), and no differences in RI. During H1, no differences were found in either RE or RI measures. During H2, differences were only observed for RI in channel 12 (HbR: adj.*p* = 0.035, *d* = 0.91), with Attention-focused group being more involved.

Post hoc comparison across sessions per group revealed following results within each channel. In channel 2, both groups displayed increasing RE (HbR: Attention-focused: adj.*p* < 0.001, *d* = −1.57; HbR: Accuracy-focused: adj.*p* < 0.001, *d* = −1.12) and decreasing RI (HbR: Attention-focused: adj.*p* < 0.001, *d* = 1.04; HbR: Accuracy-focused: adj.*p* < 0.001, *d* = 1.83)  from E1 to E3. Attention-focused group showed increasing RE (HbO: adj.*p* = 0.013, *d* = −0.79) and no change in RI transitioning from E3 to H1 sessions, while Accuracy-focused group showed decreasing RE (HbO: adj.*p* = 0.013, *d* = 0.81) and increasing RI (HbO: adj.*p* < 0.001, *d* = −1.17; HbR: adj.*p* = 0.003, *d* = −0.86). During H1 to H2, neither groups showed any significant changes in RE or RI. In channel 7, Attention-focused performers decreased RE (HbO: adj.*p* = 0.001, *d* = 1.12, HbR: adj.*p* = 0.020, *d* = 0.89) and increased RI (HbO: adj.*p* < 0.001, *d* = -–1.28; HbR: adj.*p* < 0.001, *d* = −1.36) from E1 to E3, while Accuracy-focused performers had no change in RE and decreased RI (HbO: adj.*p* = 0.001, *d* = 1.01; HbR – adj.*p* < 0.001, *d* = 1.22). Attention-focused performers increased in RE (HbO: adj.*p* = 0.001, *d* = −0.99) and decreased in level of RI (HbO: adj.*p* = 0.007, *d* = 0.80) while transitioning from E3 to H1. In contrast, Accuracy-focused performers decreased in RE (HbO: adj.*p* = 0.003, *d* = 0.82), and increased in RI (HbO: adj.*p* < 0.001, *d* = −1.24). No differences in RE were found for neither Attention-focused nor Accuracy-focused performers from H1 to H2, while decreased RE was found in Attention-focused group (HbR: adj.*p* = 0.013, *d* = 0.75). In channel 12, Attention-focused performers displayed increased RE (HbR: adj.*p* < 0.001, *d* = −1.50) and decreased RI (HbR: adj.*p* < 0.001, *d* = 1.12) from E1 to E3, while Accuracy-focused performers had no change in RE and decreased RI (HbR: adj.*p* = 0.001, *d* = 1.03). Attention-focused performers decreased in RE (HbR: adj.*p* < 0.001, *d* = 0.99) and increased in RI (HbR: adj.*p* = 0.007, *d* = -–0.79) from E3 to H1, while Accuracy-focused performers increased in RE (HbR: adj.*p* < 0.001, *d* = −0.96) and decreased in RI (HbR: adj.*p* = 0.015, *d* = 0.64). Both groups decreased in RE from H1 to H2 (HbR: Attention-focused:  = 0.046, *d* = 0.55; HbR: Accuracy-focused: adj.*p* = 0.046, *d* = 0.50), and increased their level of RI (HbR: Attention-focused: adj.*p* = 0.007, *d* = −0.74; HbR: Accuracy-focused: adj.*p* = 0.018, *d* = −0.58).

Significant interaction effects of Group and Session were observed on RE and RI measures extracted from behavioral (Adaptive target find score) and fNIRS measures from channel 2 (RE—HbO: $\chi^{2}$(*8*) = 31.43, *p* < 0.001; HbR: $\chi^{2}$(*8*) = 65.63, *p* < 0.001; RI—HbO: $\chi^{2}$(*8*) = 70.22, *p* < 0.001; HbR: $\chi^{2}$(*8*) = 29.46, *p* < 0.001) (see first column in Fig. 6). Post hoc analysis between groups per session revealed significant differences in RE for only E3 (HbR: adj.*p*= 0.046, *d* = −1.07) and RI for only H1 (HbO: adj.*p* = 0.049, *d* = −1.25), with higher RE and RI observed during these sessions by Accuracy-focused group. Post hoc analysis from E1 to E3 per group revealed significant increases in RE in both groups (HbR: Attention-focused: adj.*p* < 0.0*01*, *d* = −1.39; HbR: Accuracy-focused: adj.*p* < 0.001, *d* = −1.46), with increases in RI for Attention-focused group (HbO: adj.*p* = 0.049, *d* = −0.65) and decreases for Accuracy-focused group (HbR: adj.*p* < 0.001, *d* = 1.32). Transitioning from E3 to H1 resulted in decreased RE in Accuracy-focused group (HbO: adj.*p* = 0.016, *d* = 0.86) and no change in Attention-focused group, while RI decreased in Attention-focused group (HbO: adj.*p* = 0.011, *d* = 0.83) and increased in Accuracy-focused group (HbO: adj.*p* < 0.001, *d* = −1.43). Lastly, only RE changes were observed in Accuracy-focused group from H1 to H2 (HbR: adj.*p* = 0.031, *d* = 0.69), with significant decreases observed.

## Discussion

We evaluated skill acquisition and transfer in novice operators during training on a realistic dual task using behavioral and fNIRS measures. The results showed that assessing performance and mental load as a function of both task and individual characteristics provided further insight into changes in performance and mental load during multitask training. Specifically, both performance and mental load results during skill acquisition sessions revealed that individuals preferred to focus on improving only on one of the two tasks (scan or target find). This preference was maintained during transfer sessions. However, brain activity measures revealed that individuals who focused on scan task were able to begin concentrating on target find task by the end of skill acquisition phase. These findings were supported by relative efficiency and relative involvement measures that were utilized to assess the interaction between performance and mental load.

In addition to task characteristics, investigators have explored ways to include individual characteristics as a factor while evaluating multitask effects on performance [5, 6, 8]. In line with these studies, our results indicate that inclusion of individual differences (Group) not only improved goodness of fit (see Table 1) but also indicated significant interactions between Group and Session and significant post hoc comparisons. These individual differences can arise due to intrinsic, contextual, strategic, personality, or preference factors [11–14]. Firstly, intrinsic variability arises from differences in age, gender, and handedness, along with other participant-related attributes and experiences, while contextual variability is driven by familiarity with the task and the environment. According to cognitive load theory, intrinsic and contextual factors interact to make up the “intrinsic load” [2]. We believe the differences observed are not a result of these factors, because (1) subjects recruited in this study were all right-handed, were of similar age, and were equally distributed within each Group, and (2) prior to engaging with the task, all individuals indicated that they had limited simulator experience and had no knowledge regarding UAS operator tasks. In addition to having a similar level of expertise, all individuals underwent the same tasks, therefore suggesting contextual variability may not be the reason behind the observed individual differences. Secondly, strategic variability, also termed ‘germane load’ according to cognitive load theory, emerges when an individual adopts a strategy that best matches their expectations [2]. Our performance data strongly indicate the effect of strategy. Specifically, evaluation of adaptive target find score during skill acquisition (across easy sessions) displayed that some individuals improved, while others worsened. Furthermore, those who improved in their adaptive target find score showed decreased scan performance across easy sessions. This finding indicates that individuals prioritized tasks differently. In other words, individuals preferred to focus on one task over the other. Prioritization during multitasking is common strategy employed to preserve performance on at least one of the tasks [15–20]. Such a strategy is commonly referred to as serial or blocked processing, where individuals focus on one task before moving to the other. Other strategies include parallel or adaptive (shifting between serial and parallel) processing. Although which strategy is ideal is dependent on other individual characteristics, a consensus among researchers is that experts are able to use adaptive processing strategy, where an individual can maintain a balance between minimizing between-task interferences and mental effort, via increasing serial and parallel processing, respectively [51]. Therefore, we can assume that individual differences observed in our study are most likely due to strategy, particularly the strategy derived from preference.

Prior to interpreting effects of individual differences or training on brain activity measures, we first should understand what type of PFC response is elicited by UAS operators during execution of search and surveillance task. Our results depicted significant brain activity changes within most channels for both HbO and HbR biomarkers (see Fig. 5A). This effect being over all the regions (global effect) is likely due to the nature of the task requiring execution and coordination among multiple cognitive processes. This finding is in agreement with previous studies, which have indicated that brain activity during complex tasks is not localized to one specific PFC area [26, 28, 31, 36, 52, 53]. However, post hoc results indicated significant differences primarily within channels 2, 7, 11, 12, and 14. These results suggest that even though most of the PFC was recruited to perform the task, that stronger activity was observed in the task-relevant areas. Specifically, fNIRS studies have shown that channel 2 is approximately measuring from the left dorsolateral prefrontal cortex (LDLPFC), which is reported to be involved in spatial working memory or recognizing specific features and task setting [34, 42, 54–56]. Alternatively, channels 11, 12, and 14 are the measures from the right anterior medial PFC (RAMPFC), which is known to be involved with attentional control [34, 42, 55]. Activity within the LDLPFC and RAMPFC regions implies that the search and surveillance task employed in this study taxes attention and spatial working memory processes [57]. Activations within these channels and regions are in line with other similar fNIRS and fMRI studies evaluating brain activity during spatial navigation tasks [28, 39, 58–60]. Lastly, channel 7 is overlaid on top of the left anterior medial PFC (LAMPFC) and has been shown by fMRI studies to be involved with task switching [61, 62]. Activity within this region may suggest executive controls involvement in enabling simultaneous engagement in scan and target find tasks.

Without accounting for individual characteristics, our results from skill acquisition phase indicated significant decreases in mental effort within LDLPFC and RAMPFC (see Additional file 1: Table S1). These results support neural plasticity and practice theory, which states that practice is effective in decreasing brain activity intensity within attentional and control areas [22, 30, 33, 52, 59, 63]. Secondly, our results from transfer phase showed significant increases within LAMPFC and RAMPFC. These results are supported by cognitive workload theory, that states that mental load increases with increases in task demands [22, 28, 52, 53, 57]. Inclusion of individual differences significantly improved model fitness of mean fNIRS measures (see Table 2). Similar, effects on brain activity changes using individual characteristics have been reported by other researchers [7, 27, 55, 58, 60–62, 64–67]. The improvement was especially observed for HbR measures, which has been shown to be a more sensitive measure of cognitive activity than HbO for this particular task [45]. Furthermore, inclusion of these individual differences has provided further insight into interaction between performance and mental effort changes during multitasking training.

### Skill acquisition

Both Attention-focused and Accuracy-focused groups displayed a decrease in brain activity within RAMPFC and LDLPFC areas. These results align with the ones reported when individual differences were not accounted for. However, activity within LAMPFC increased in the Attention-focused group, while it decreased in the Accuracy-focused group. These results were not observed when individual differences were not accounted for. Since LAMPFC is involved in task switching, a possible interpretation could be that the Attention-focused group was switching their strategy from serial to parallel task processing or using adaptive processing. Similar brain activity changes associated with differences in strategy have been shown in previous studies [68–70]. These studies have identified that this shift in processing may occur because of expertise development. However, level of neural activation should be interpreted considering behavioral measures [26, 31, 32]. Interpreting brain activity changes in RAMPFC and LDLPFC results considering scan performance indicated that the Attention-focused group was more efficient at the scan task, while the Accuracy-focused group was more efficient at target find task. Furthermore, although the Attention-focused group did not improve in target find performance by the end of skill acquisition phase, their relative involvement measures indicated that they were involved in both tasks by the end of the phase. These results further support a shift in processing strategy from a serial to parallel or adaptive processing with practice. These involvement results are not only further supported by the RE and RI changes in LDLPFC (see Figure first two columns in Fig. 6) but are also supported by the results where scan and fNIRS measures changed as a function of Group, Session, and Adaptive target find score. Specifically, the results show that scan measures decreased with target find during easy session 1 and increased during easy session 3 (see Additional file 1: Figure S4A), while brain activity increased in easy session 1 and 3 when target was found (see Additional file 1: Figure S4B). Alternatively, analysis of this task also supports the fact that the Accuracy-focused group was only involved in the target find task. Specifically, the results showed that the scan measures increased with target find during easy session 1 and decreased during easy session 3 (see Additional file 1: Figure S4A). This shift from negative to positive association suggests that the performers either stopped scanning after they found the target or were aimlessly wandering. The association between adaptive target find score and fNIRS measures from LDLPFC moved from a positive to negative, indicating that the subjects went from using more to fewer resources with practice, while finding targets (see Additional file 1: Figure S4B). Such associations were not observed in the Attention-focused group, indicating that even though they were task switching, they needed additional practice to improve target find performance. In summary, Attention-focused group was efficient at acquiring scan skills and remained involved in both tasks, while Accuracy-focused was efficient and involved in only acquiring target find skills.

### Transfer

The behavioral results indicate that introduction to a task of higher load resulted in maintenance of scan performance by both Attention-focused and Accuracy-focused groups. fNIRS results from Attention-focused group displayed increased activity within RAMPFC, decreased activity within LAMPFC, and no change in LDLPFC, while Accuracy-focused group demonstrated decreased activity within RAMPFC and increased activity within LDLPFC and LAMPFC. These changes are different from the findings when individual differences were not accounted for. Although these results indicate that an increase in task load led to recruitment of more neural resources to maintain similar behavioral outcomes as that observed during performance of the easy tasks, the recruitment of neural resources varied across PFC region and group. Specifically, even though Attention-focused group had begun changing their strategy from serial to parallel processing by the end of skill acquisition phase, increases in task load reverted their strategy back to prioritizing the scan task. Similar shifting in strategy due to increased task load has been observed in other studies [4, 17–19]. Accuracy-focused group continued to prioritize the target find task. Assessment of relative efficiency and relative involvement measures provide further insight. In particular, the Attention-focused group decreased in efficiency and remained involved in scan tasks, while ignoring the target find task. These results validate that the Attention-focused group needed more practice on the target find task during easy conditions before being able to transfer the skills to the hard condition. Additionally, the Attention-focused group displayed a decrease in relative efficiency across hard sessions, while their relative involvement remained high. This further supports the different prioritization strategies used by the two groups. Unlike the Attention-focused group, the Accuracy-focused group was relatively efficient in the scan task, and they were not when performing the target find task. However, they were relatively involved in the target find task and not relatively involved in the scan task. As previously described the target find task is a secondary task to the primary scan task, which means that improvement in scan task performance should enable improvement in the target find task. Based on this presumed connection, the Accuracy-focused group could be zooming in further to accommodate for the lack of visibility in the hard condition; therefore, they may have utilized scan task performance as way of completing the target find task. This could be the reason why the Accuracy-focused group had increased activity in LAMPFC and LDLPFC, but not in RAMPFC. Lastly, the Accuracy-focused group showed no change in relative efficiency or relative involvement across hard conditions. A possible reason for this could be that they gave up or that they were mentally fatigues or overloaded. In summary, Attention-focused group was involved in transferring their scan skills, while the Accuracy-focused group was involved in transferring their target find skills.

### Limitation

Despite the promising methodology and results, the study results are subject to a few limitations. This study recruited a limited sample size. Specifically, the Attention-focused group had *N* = 6 individuals, while Accuracy-focused group had *N* = 7. Therefore, the findings reported here are preliminary in nature and future studies when a larger sample cohort is needed, especially when investigating individual differences. fNIRS signals are influenced by extracerebral and systemic activity. We utilized “(0 + ShortSDS | ID)” to account for global extracerebral and systemic activity. Although the inclusion of this factor significantly improved model fitness (see Additional file 1: Table S2), the factor does not remove task-evoked extracerebral and systemic activity [71–73]. Therefore, future studies will need to incorporate signal processing techniques, such as least squares adaptive filters, Kalman filter, and state-space model-based methods, to improve removal of task-evoked and non-evoked extracerebral and systemic activities [74]. The brain activity results must be interpreted with caution, as not all areas of the brain that are involved with the UAS operator search and surveillance task could be measured with fNIRS technology employed in this study. Mean fNIRS measures were calculated from fifteen seconds after onset and before the end of a subarea, leading to an average over 90 s. Studies have indicated that averaging over trials longer than 60 s may include unrelated cortical activity and have suggested parsing of the fNIRS time series into small time segments before averaging [43]. Future studies will incorporate analysis of the temporal changes in brain activity while performing the task of interest. Such analyses will allow for examination of whether scan performance remained similar or depreciated after finding a target and in turn validate the results regarding strategy used during task interleaving. Personality factors, such as cooperation, motivation, habituation, awareness, and stress, are known to effect multitasking; therefore, future studies should include survey between sessions to assess these effects [7]. To further investigate difference in strategy, future studies should compare dual task results against single tasks within same subjects and should experimentally induce different task interleaving processes.

In conclusion, our study provides a unique insight into individual differences through neural and behavioral measures while we analyze human performance in a dual and ecologically valid task. We demonstrated that including individual differences as a factor can enhance assessment of skill acquisition and transfer during multitask training. This study contributes to the existing literature and reports that brain activity changes acquired via fNIRS are sensitive to changes in task demands and that complex task execution elicits recruitment of resources within multiple regions of the PFC. We posit that the changes in cortical activity, particularly within left anterior medial prefrontal cortex region, could be associated with task switching. Our results support previous findings that task practice results in an improvement in behavioral performance metrics and a reduction in the level of brain activity changes. Lastly, our results highlight that integrated behavioral performance and brain activation assessments of relative efficiency and relative involvement are improved metrics for describing skill acquisition and transfer.

## Supplementary Information

**Additional file 1: Figure S1.** Example scan and target find performance from a particular Subject, Session, and Subarea. A. Raw FOV polygons overlaid on task area. B. FOV polygons that had Bottom Max Size less than 750 and FOV Area Ratio less than 0.50 result in scan, not scan, and over scan ratios of 0.57, 0.13, and 0.29, respectively. During this subarea the target was not found.** Figure S2.** Changes in behavioral measures as a function of Session and Adaptive target find score per performance group. Attention-focused performers (N = 6) and Accuracy-focused performers (N = 7). Plotted points reflect mean. Easy 1 – E1, Easy 2 – E2, Easy 3 – E3, and Hard 1 – H1 and Hard 2 – H2.** Figure S3.** Post hoc comparisons between sessions per group across all channels and fNIRS measures. Comparisons consisted of easy session 1 – easy session 3 (across easy), easy session 3 – hard session 1 (between easy and hard), and hard session 1 and hard session 2 (across hard). Only effects (Cohen’s d) associated with significant (a < 0.05) comparisons were plotted. Cohen’s d of 0.2 is considered a small effect, while 0.5 and 0.8 represent medium and large effects, respectively. If Cohen’s d is negative for HbO and positive for HbR, then this indicates that the activity increased in the second term of the comparison. For example, in Attention-focused performers, channel 13 displayed higher activity in easy session 1 than easy session 3 (HbO d = 1.61; HbR d = -0.87), while channel 7 displayed higher activity in easy session 3 than easy session 1 (HbO d = -0.74; HbR d = 0.92).** Figure S4.** Association between Adaptive target find score and behavioral or fNIRS measures across easy sessions per group. Dark line represents smoothed conditional mean or regression line, while shaded regions represent confidence interval of 0.95. Table S1. Post Hoc comparisons between Sessions of model that did not include individual differences. Cohen’s $d$ of 0.2 is considered a small effect, while 0.5 and 0.8 represent medium and large effects, respectively. If Cohen’s $d$ is negative for HbO and positive for HbR, then this indicates that the activity increased in the second term of the comparison. For example, in channel 1 higher activity is observed in easy session 1 than easy session 3 for HbO and HbR. Table S2. Effect of systemic activity on fNIRS measures. Comparing model with 1 + (0+ShortSDS|ID) term against 1+ (1|ID).

## Data Availability

The data that support the findings of this study are available from the corresponding authors, PR and KI, upon request.
