# Supplementary material for: Individual differences in skill acquisition and transfer assessed by dual task training performance and brain activity
Source: Brain Inform. 2022 Apr 2;9(1):9. doi: 10.1186/s40708-022-00157-5 (PMC8976865; doi:10.1186/s40708-022-00157-5)
Supplement: Supplementary file 1 — Additional file 1: Figure S1. Example scan and target find performance from a particular Subject, Session, and Subarea. A. Raw FOV polygons overlaid on task area. B. FOV polygons that had Bottom Max Size less than 750 and FOV Area Ratio less than 0.50 result in scan, not scan, and over scan ratios of 0.57, 0.13, and 0.29, respectively. During this subarea the target was not found. Figure S2. Changes in behavioral measures as a function of Session and Adaptive target find score per performance group. Attention-focused performers (N = 6) and Accuracy-focused performers (N = 7). Plotted points reflect mean. Easy 1 – E1, Easy 2 – E2, Easy 3 – E3, and Hard 1 – H1 and Hard 2 – H2. Figure S3. Post hoc comparisons between sessions per group across all channels and fNIRS measures. Comparisons consisted of easy session 1 – easy session 3 (across easy), easy session 3 – hard session 1 (between easy and hard), and hard session 1 and hard session 2 (across hard). Only effects (Cohen’s d) associated with significant (a < 0.05) comparisons were plotted. Cohen’s d of 0.2 is considered a small effect, while 0.5 and 0.8 represent medium and large effects, respectively. If Cohen’s d is negative for HbO and positive for HbR, then this indicates that the activity increased in the second term of the comparison. For example, in Attention-focused performers, channel 13 displayed higher activity in easy session 1 than easy session 3 (HbO d = 1.61; HbR d = -0.87), while channel 7 displayed higher activity in easy session 3 than easy session 1 (HbO d = -0.74; HbR d = 0.92). Figure S4. Association between Adaptive target find score and behavioral or fNIRS measures across easy sessions per group. Dark line represents smoothed conditional mean or regression line, while shaded regions represent confidence interval of 0.95. Table S1. Post Hoc comparisons between Sessions of model that did not include individual differences. Cohen’s [file 40708_2022_157_MOESM1_ESM.docx]

**Additional file 1**


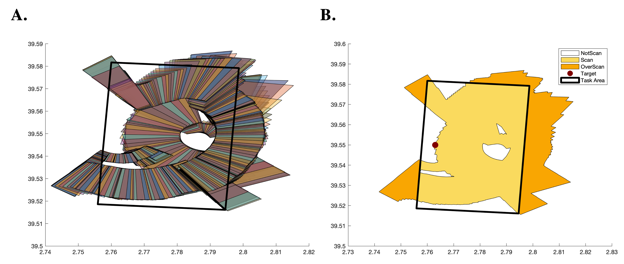


**Additional file 1: Figure S1. Example scan and target find performance from a particular Subject, Session and Subarea. A.** Raw FOV polygons overlayed on task area. **B.** FOV polygons that had Bottom Max Size less than 750 and FOV Area Ratio less than 0.50 result in scan, not scan, and over scan ratios of 0.57, 0.13, and 0.29, respectively. During this subarea the target was not found.


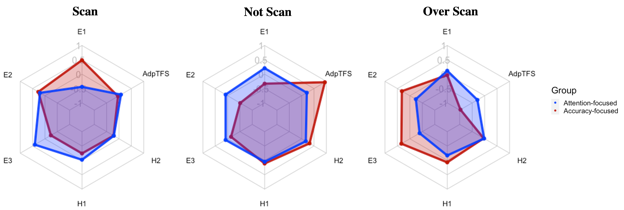


**Additional file 1: Figure S2. Changes in behavioral measures as a function of Session and Adaptive target find score per performance group.** Attention-focused performers (N = 6), and Accuracy-focused performers (N = 7). Plotted points reflect mean. Easy 1 – E1, Easy 2 – E2, Easy 3 – E3, and Hard 1 – H1 and Hard 2 – H2.

##
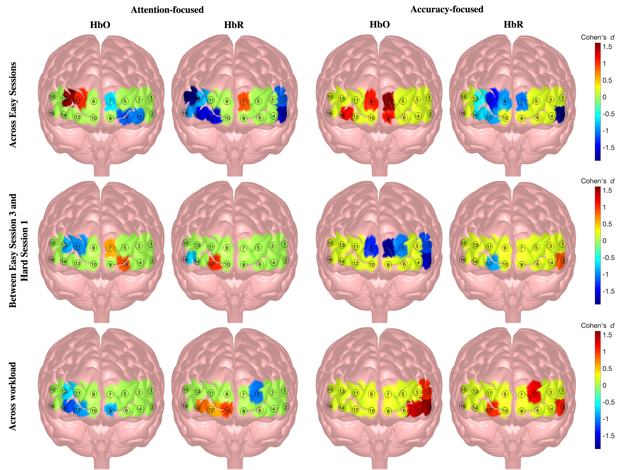


## **Additional file 1: Figure S3. Post hoc comparisons between Sessions per Group across all channels and fNIRS measures.** Comparisons consisted of easy session 1 – easy session 3 (across easy), easy session 3 – hard session 1 (between easy and hard), and hard session 1 and hard session 2 (across hard). Only effects (Cohen’s d) associated with significant (a < 0.05) comparisons were plotted. Cohen’s d of 0.2 is considered a small effect, while 0.5 and 0.8 represent medium and large effects, respectively. If Cohen’s d is negative for HbO and positive for HbR, then this indicates that the activity increased in the second term of the comparison. For example, in Attention-focused performers, channel 13 displayed higher activity in easy session 1 than easy session 3 (HbO d = 1.61; HbR d = -0.87), while channel 7 displayed higher activity in easy session 3 than easy session 1 (HbO d = -0.74; HbR d = 0.92).

**
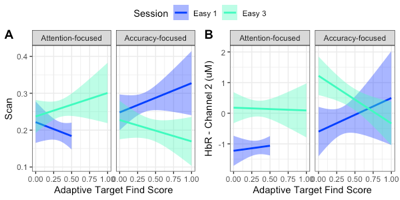
**

**Additional file 1: Figure S4. Association between Adaptive target find score and behavioral or fNIRS measures across easy sessions per Group.** Dark line represents smoothed conditional mean or regression line, while shaded regions represent confidence interval of 0.95.

**Additional file 1: Table S1. Post Hoc comparisons between Sessions of model that did not include individual differences.** Cohen’s $d$ of 0.2 is considered a small effect, while 0.5 and 0.8 represent medium and large effects, respectively. If Cohen’s $d$ is negative for HbO and positive for HbR, then this indicates that the activity increased in the second term of the comparison. For example, in channel 1 higher activity is observed in easy session 1 than easy session 3 for HbO and HbR.

|  |  | E1 - E3 | | E3 - H1 | | H1 - H2 | |
| --- | --- | --- | --- | --- | --- | --- | --- |
| **Channel** | **Biomarker** | **p value** | **Cohen's *d*** | **p value** | **Cohen's *d*** | **p value** | **Cohen's *d*** |
| 1 | HbO | 0.250 | 0.29 | 0.020 | -0.59 | 0.250 | 0.22 |
|  | HbR | 0.149 | -0.43 | 0.059 | -0.48 | 0.536 | 0.13 |
| 2 | HbO | 0.535 | 0.13 | 0.747 | -0.14 | 0.747 | -0.06 |
|  | HbR | <0.001 | -1.68 | 0.420 | -0.18 | <0.001 | 0.68 |
| 3 | HbO | 0.747 | 0.23 | 0.011 | -0.63 | <0.001 | 0.72 |
|  | HbR | 0.775 | -0.06 | 0.430 | 0.19 | 0.430 | -0.16 |
| 4 | HbO | 0.212 | -0.32 | 0.381 | 0.17 | 0.007 | 0.54 |
|  | HbR | 0.835 | -0.21 | 0.381 | 0.17 | 0.012 | -0.48 |
| 5 | HbO | 0.857 | 0.04 | 0.002 | -0.66 | 0.816 | -0.04 |
|  | HbR | 0.934 | 0.11 | 0.720 | 0.08 | 0.720 | -0.07 |
| 6 | HbO | 0.046 | -0.57 | 0.006 | -0.61 | 0.527 | -0.12 |
|  | HbR | 0.895 | 0.03 | 0.047 | -0.46 | 0.704 | -0.07 |
| 7 | HbO | 0.214 | 0.29 | 0.783 | -0.06 | 0.783 | -0.07 |
|  | HbR | 0.704 | -0.12 | 0.002 | -0.66 | 0.720 | -0.06 |
| 8 | HbO | 0.007 | 0.57 | 0.854 | -0.18 | 0.983 | 0.00 |
|  | HbR | 0.074 | -0.32 | 0.866 | -0.03 | 0.866 | 0.16 |
| 9 | HbO | 0.212 | 0.28 | 0.867 | 0.04 | 0.202 | -0.28 |
|  | HbR | 0.004 | -0.54 | 0.510 | 0.12 | 0.109 | 0.29 |
| 10 | HbO | 0.720 | 0.08 | 0.775 | -0.13 | 0.775 | -0.13 |
|  | HbR | 0.249 | -0.25 | 0.835 | -0.05 | 0.835 | 0.04 |
| 11 | HbO | 0.010 | 0.54 | 0.054 | -0.51 | 0.969 | 0.01 |
|  | HbR | <0.001 | -0.98 | 0.454 | -0.31 | 0.454 | -0.21 |
| 12 | HbO | 0.854 | 0.04 | 0.083 | 0.39 | 0.006 | -0.56 |
|  | HbR | <0.001 | -1.19 | 0.023 | -0.45 | 0.001 | 0.65 |
| 13 | HbO | 0.235 | 0.36 | 0.717 | 0.07 | 0.717 | -0.11 |
|  | HbR | <0.001 | -0.72 | 0.457 | -0.14 | <0.001 | 0.74 |
| 14 | HbO | 0.018 | 0.51 | 0.767 | -0.05 | <0.001 | 0.71 |
|  | HbR | <0.001 | -1.08 | 0.038 | 0.42 | <0.001 | -0.84 |
| 15 | HbO | 0.854 | 0.14 | 0.627 | -0.09 | 0.200 | 0.27 |
|  | HbR | <0.001 | -0.87 | 0.619 | -0.09 | 0.094 | 0.32 |
| 16 | HbO | 0.866 | 0.03 | 0.924 | 0.02 | 0.924 | 0.09 |
|  | HbR | <0.001 | -0.78 | 0.282 | -0.24 | 0.518 | 0.11 |

**Additional file 1: Table S2. Effect of systemic activity on fNIRS measures.** Comparing model with 1 + (0+ShortSDS|ID) term against 1+ (1|ID).

| **Channel** | **HbO** | | **HbR** | |
| --- | --- | --- | --- | --- |
|  | $\boldsymbol{\chi}^{\boldsymbol{2}}$**(4)** | **p value** | $\boldsymbol{\chi}^{\boldsymbol{2}}$**(4)** | **p value** |
| 1 | 102.72 | <0.001 | 122.65 | <0.001 |
| 2 | 96.10 | <0.001 | 49.56 | <0.001 |
| 3 | 183.42 | <0.001 | 66.53 | <0.001 |
| 4 | 115.49 | <0.001 | 132.47 | <0.001 |
| 5 | 80.35 | <0.001 | 17.45 | <0.001 |
| 6 | 46.74 | <0.001 | 63.15 | <0.001 |
| 7 | 133.87 | <0.001 | 38.02 | <0.001 |
| 8 | 141.76 | <0.001 | 66.31 | <0.001 |
| 9 | 207.79 | <0.001 | 49.48 | <0.001 |
| 10 | 190.45 | <0.001 | 27.93 | <0.001 |
| 11 | 217.47 | <0.001 | 10.22 | 0.001 |
| 12 | 152.96 | <0.001 | 52.60 | <0.001 |
| 13 | 170.28 | <0.001 | 12.56 | <0.001 |
| 14 | 185.11 | <0.001 | 70.46 | <0.001 |
| 15 | 123.93 | <0.001 | 57.79 | <0.001 |
| 16 | 214.49 | <0.001 | 61.79 | <0.001 |
